# Turning Text into Research Networks: Information Retrieval and Computational Ontologies in the Creation of Scientific Databases

**DOI:** 10.1371/journal.pone.0027499

**Published:** 2012-01-03

**Authors:** Flávio Ceci, Ricardo Pietrobon, Alexandre Leopoldo Gonçalves

**Affiliations:** 1 Department of Knowledge Engineering and Management, Federal University of Santa Catarina, Santa Catarina, Brazil; 2 Associate Professor and Vice Chair, Department of Surgery, Duke University Medical Center, Durham, North Carolina, United States of America; 3 Director of Research on Research Group, Duke University, Durham, North Carolina, United States of America; University of Chicago, United States of America

## Abstract

**Background:**

Web-based, free-text documents on science and technology have been increasing growing on the web. However, most of these documents are not immediately processable by computers slowing down the acquisition of useful information. Computational ontologies might represent a possible solution by enabling semantically machine readable data sets. But, the process of ontology creation, instantiation and maintenance is still based on manual methodologies and thus time and cost intensive.

**Method:**

We focused on a large corpus containing information on researchers, research fields, and institutions. We based our strategy on traditional entity recognition, social computing and correlation. We devised a semi automatic approach for the recognition, correlation and extraction of named entities and relations from textual documents which are then used to create, instantiate, and maintain an ontology.

**Results:**

We present a prototype demonstrating the applicability of the proposed strategy, along with a case study describing how direct and indirect relations can be extracted from academic and professional activities registered in a database of curriculum vitae in free-text format. We present evidence that this system can identify entities to assist in the process of knowledge extraction and representation to support ontology maintenance. We also demonstrate the extraction of relationships among ontology classes and their instances.

**Conclusion:**

We have demonstrated that our system can be used for the conversion of research information in free text format into database with a semantic structure. Future studies should test this system using the growing number of free-text information available at the institutional and national levels.

## Introduction

The volume of Web-based, free-text documents containing information on science and technology is growing at an increasing rate [1]. Since these documents are not immediately processable by computers in their original format, it takes longer and might lead to pressure from academic institutions, governments and industry to turn this raw data into useful information. Although computational ontologies represent a significant improvement in representing this massive amount of information, their creation, instantiation and maintenance continues to rely on manual methods [2]. As a result, the process to turn free text into discrete data sets is slowed down, ultimately delaying the acquisition of valuable information out of the data.

Computational ontologies address the problem of data representation for systems that are consistently changing over time [3]. For example, imagine a data set containing information about a group of researchers from a given university, including their names, institutions, publications, patents, and classes they teach. This information changes over time, meaning that every year each faculty is adding more of each of these academic products. Each of these categories also have relations among them, in the sense that a given researcher could be an author in a paper, have another researcher as a co-author, and be a faculty member at a given institution.

Ontologies include controlled vocabularies which provide structured definitions and reasoning to terms from a particular domain, and also allow inferences once the system is instantiated [4]. For example, the ontology could say that “researchers “Alex” and “Flavio” are co-authors on a paper called “Extracting content-rich information …,” that “co-authors from the same institution are part of a research team.” From this set of information, the ontology would be able to infer that “Alex and Flavio are part of the same research team.” While regular relational database systems like Microsoft Access [5], last accessed February 2011) or Oracle [6] – represent data that does not constantly change in structure, computational ontologies can be dynamically updated as the underlying data changes over time. This dynamic aspect is achieved by representing data using the concept of triples, or the organization of subject-predicate-object or class-relation-class structures [7], [8]. For example, if we consider two researchers as instances of a class called “author” and the action “citing” as representing a relationship, these three elements could form a triple of the form “author A” “cites” “author B.” The flexibility of computational ontologies relies on how easily they can be changed, since to modify its structure one simply has to add a triple. A problem secondary to this scalability is that in order to add a triple one still has to make use of ontology engineering processes to determine what the class and relationship should contain. Currently, most ontology engineering processes advocate manual creation of classes and relations and, therefore, lack scalability [2].

When it comes to processing scientific information from free text, the challenges reported earlier increase for at least two reasons. First, the volume of free text is massive and growing at an increasing rate, PubMed alone having grown by over 700,000 new abstracts in the last 12 months [9]. Second, the quality of the manually created ontologies is difficult to evaluate and therefore inconsistent, since the structure of the ontology has to change as additional free text is processed [2]. Several previous publications have partially addressed this issue through methods attempting to automate the process of creation, maintenance, and instantiation of computational ontologies. Although a number of automated, non-supervised algorithms have been developed [10], automated algorithms still present a significant misclassification rate, especially when facing specialized terms that have not been extensively studied.

The overall objective of this article is therefore to present a method to semi-automatically construct, maintain, and instantiate scientific ontologies. The method makes use of a novel combination of gazetteers for named-entity recognition, the LINGO algorithm for labeling cluster instances, and social network data sets for semi-automated discovery of classes and relations [11]. Our study is described in three main sections. First, we present the solution architecture with details on the methods and technology. Second, a case study, where the architecture is placed in context of an example. Finally, an experimental section presents results of the solution architecture used on a large scale data set.

## Solution Architecture

### Overview

The first stage comprises classic named-entity recognition using gazetteers, where elements are tagged and clusterized from the original text. These tags are automatically attributed to generic classes using the LINGO algorithm [11], thus reducing human intervention. In subsequent validation stages, a verification is conducted to determine whether the entity is valid for the proposed class domain, making use of the knowledge base Wikipedia. For an entity to be classified as valid, the term should be recognized in the Wikipedia database. If the term is not recognized, then a search is carried out for for similar terms. The top ten most similar terms are searched in the index and, if they exist, they are added to the list of valid terms. Otherwise they are considered invalid. These entities are then visually inspected by the knowledge engineer, who designs a new classification, suggests the use of new classes where the algorithm did not previously classify an element, or simply discards the elements. In addition, the automated classification is verified to ensure an accurate association between instances and their proposed classes. Of importance, our method does not assume the pre-existence of a domain ontology, thus characterizing our method as semi-automated and incremental. The choice of a semi-automated method allowed the curation to be performed on a selected portion of the database in an iterative manner, where the knowledge engineers manage entities as the need arises. Some of the key technologies that make our approach unique, namely the use of gazetteers for named-entity recognition, the clustering LINGO algorithm method for labeling cluster instances, and social network data sets for semi-automated discovery of classes and relations are further described in the following sections.

### Gazetteers for named-entity recognition

Named entity recognition (NER) is considered a part of information extraction, where the goal is to find and categorize sections of text into pre-established categories [12]. Specifically, named-entity recognition was initiated by the automated generation of a named-entity dictionary, known as a gazetteer. In this algorithm, we have used a number of seed words to retrieve an initial set of Web pages, later using them to acquire additional pages. For example, an initial set of city names would lead to a corresponding set of Web pages, which would lead to additional terms [13]. With the entities retrieved and classified in separate lists, the BALIE (Baseline Information Extraction) algorithm locates and compares each term, also known as token, from a given text in this dictionary BALIE is a two-module, multi-language system for information extraction from free text.

In our project, named-entity recognition is initiated by using initial seed words for each class which can be acquired from public dataset, for instance city names, or from organization database, for instance, collaborator names. It will compose a knowledge base known as gazetteer. After that, the process is incremental once instances corrected classified by the NER process and certified by the specialist will be added to the gazetteer automatically aiming to improve further results. With the gazetteer BALIE (Baseline Information Extraction) algorithm locates and compares each term, also known as token, from a given text in this dictionary.

The named-entity recognition process generated multiple instances representing the same entity. Once that process was concluded, the knowledge engineer was then responsible to tag the base entity along with the other entities that were simply associated to it. This information was stored in the knowledge base in order to refine all subsequent recognition processes.

### LINGO algorithm for labeling cluster instances

The LINGO algorithm organizes texts into hierarchical thematic clusters. This process is automated and independent from other knowledge bases, being based on the principle of singular value decomposition [12]. The LINGO algorithm is a part of the Carrot process, which is based on two major groups or processing components: Document sources, which provide the text material for further processing, and clustering algorithms themselves such as Lingo [11]. Briefly, during the first step we extracted frequent phrases as well as isolated frequent terms contained within documents. Then, singular value decomposition was used to create and decompose a term-document matrix based on frequent phrases and single terms which exceed a predefined threshold. The goal within this action was to discover latent abstract concepts represented by vectors associated with these sentences and isolated terms. As a result, each concept had a set of associated vectors which were used to assign relevant documents to the concept. The carrot clustering algorithms could be called through a number of APIs (Application programming interface) for Java.

### Social network data sets for semi-automated discovery of classes and relations

Once terms were recognized using gazetteers and appropriately clustered using LINGO, they were validated taking Wikipedia as the source of annotations. Wikipedia is a free online encyclopedia that results from an ongoing collaborative effort of volunteers [14]. In our project recognized entities were validated by searching related articles in Wikipedia while attempting to suggest possible classifications. This sequence is possible through the analysis of the class description included in the architecture ontology compared against Wikipedia articles. This sequence of technologies is formally described in Table S1.

**Figure 1:**
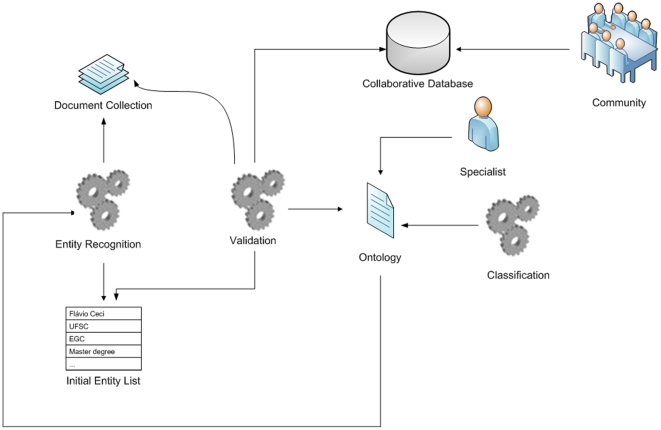
Solution architecture.

This sequence of technologies acts upon a collection of free text files, from which each instance (entity) is extracted. Each entity can be formally represented through the following vector:
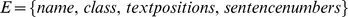
where “name” represents an instance such as academic institution (e.g., Federal University of Santa Catarina), “class” is the ontology class corresponding to the instance (e.g., institution), “text positions” is the list of positions where the entity is located, and “sentence numbers” is the list of identifiers used to label each sentence in the overall text. According to this vector, any two entities are considered to be correlated when included in a text, co-occurring in the same sentence and within a certain distance or window threshold. From this vector, we then extract distinct instances characterized as matrix indices. A matrix demonstrating the association among multiple instances is generated, with cells containing the value of the correlation coefficient among them. The system then verifies the frequency of instances contained in the index vector, generating a square matrix aligned with the vector size. Next, all matrix terms are combined to index the degree of the co-occurrence between any two terms [15]. All correlations are measured at the sentence rather than the document level. These entities are then reviewed by a knowledge engineer who will exclude the entities that are not considered relevant for the knowledge domain. The resulting validated vector is finally submitted to the correlation algorithm, which will determine the weighted correlation between entities and classes (Figure 1 and Table S2).

Given the text describing a person's curriculum in free text, entities are recognized by the system in conjunction with BALIE [13]. The first BALIE module is used for the creation of gazetters, or lists of terms that belong to a given class. The second BALIE module uses simple heuristics to identify and classify the entities in accordance to the context in which they were inserted so that it can assist in the resolution of ambiguities. This module uses the classification algorithms from the algorithm library for Weka data mining tool [16]. Since version 1.8 does contain neither terms in Portuguese nor the mapping of knowledge areas, organization and people names and acronyms organized as gazetters, we extended BALIE through its Java API (application programming interface) as well as the manual generation of gazetter for knowledge areas, organizations and people from the Lattes Platform. Details regarding this customization are provided in the Appendix S1.

The correlation between entities is calculated based on the co-occurrence frequency divided by the average in the window measurement between entities:
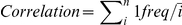
where freq is the frequency of entity occurrence (joint frequency) in a sentence, also representing the average window. A window is defined as the quantity of terms occurring between the entities of a sentence. For example, in the sentence “Flavio Ceci completed his undergraduate degree in Computer Sciences,” the window between the entities “Flavio Ceci” and “Computer Sciences” equals 4 since there are four terms between the two entities.

The average window (j) is calculated through the formula
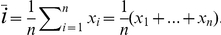
In the above example the frequency of entities (*n*) is 1, since the terms “Flávio Ceci” and “Computer Sciences” only occur once and the window (*x_i_*) has a value of 4, since the terms “Flávio Ceci” and “Computer Sciences” have four words between them within the original sentence. Applying the previous formula to this case, we would
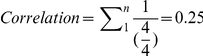
have. With the matrix in place, the most relevant entities and their correlation degree are presented to a human user. This result assists in the maintenance process, since it represents possible instances for each class and corresponding relations. Terms that are not part of this domain or were not relevant were manually excluded. All remaining entities are processed through the correlation algorithm.

This architecture can be used for both the maintenance of an existing ontology as well as the creation of a new ontology. The structure can be visualized in a graph network to facilitate decision support in the maintenance and creation activities. In order to execute element identification, we used the BALIE framework version 1.8 [13].

### Case study

We used a data set corresponding to the description Federal University of Santa Catarina (Brazil) hosted within the Lattes Platform database [17]. The data set contains information regarding academic activities from faculty and students in free text, including professional activities, knowledge areas, and institutional information. An example that will serve as the basis for this case study is presented in Table S2.

Table 1 presents a summary of the main results from the test example. Individual results are presented all in lower case text as a consequence of pre-processing. Further automation could be obtained, for example, by setting minimum correlation threshold values required to accept two classes to be considered as related.

**Table 1:** Example of degree of correlation among the instance “alexandre” and other instances and respective classes.

*Instances (classes)*	*alexandre (person)*
computer sciences (a*rea*)	0.125
production engineer (area)	0.0263
knowledge engineer (a*rea*)	0.029
blumenau university (*organization*)	0.083
stela institute (*organization*)	0.1

**Figure 2:**
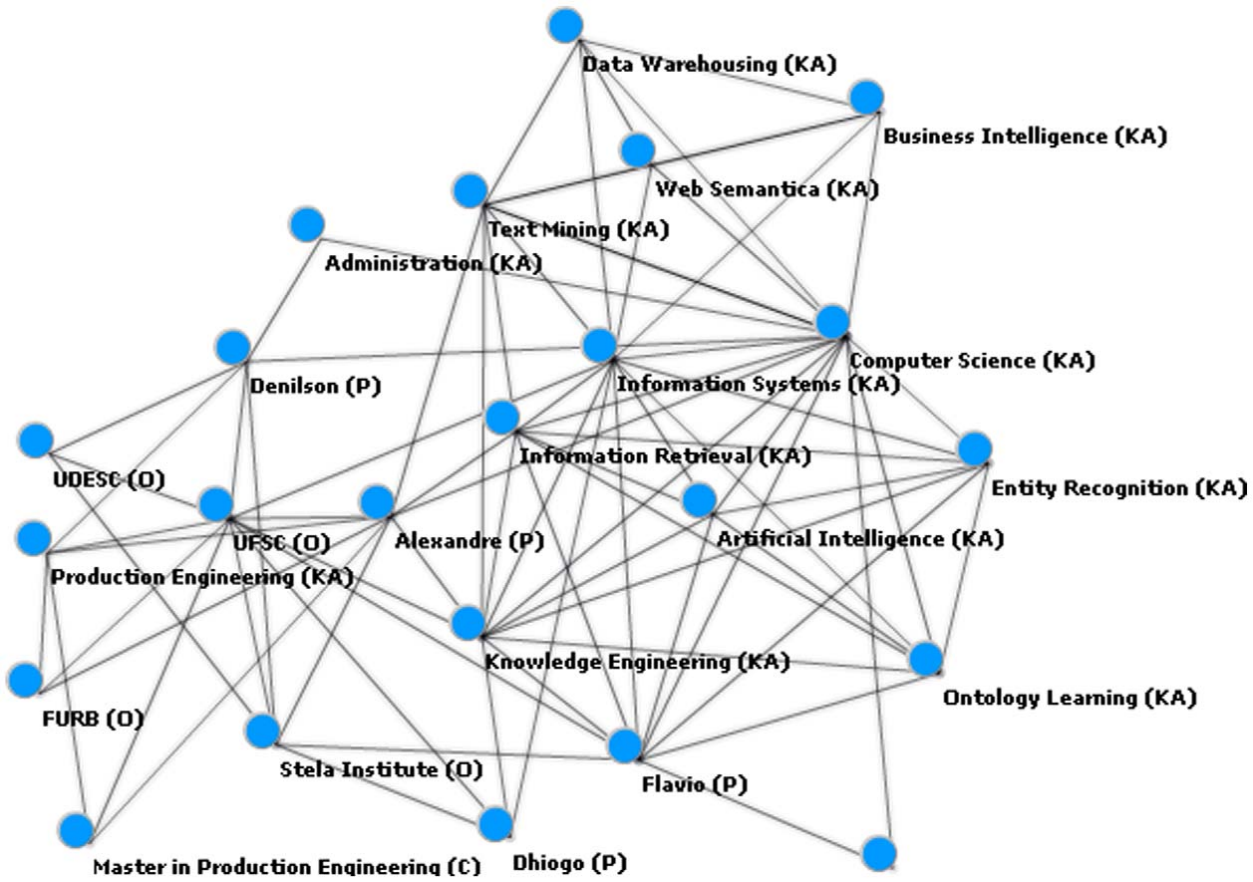
Resulting instances (classes) and their relations.

To facilitate visualization to better demonstrate relations among instances, a graphical network representation can be created (Figure 2).

**Figure 3:**
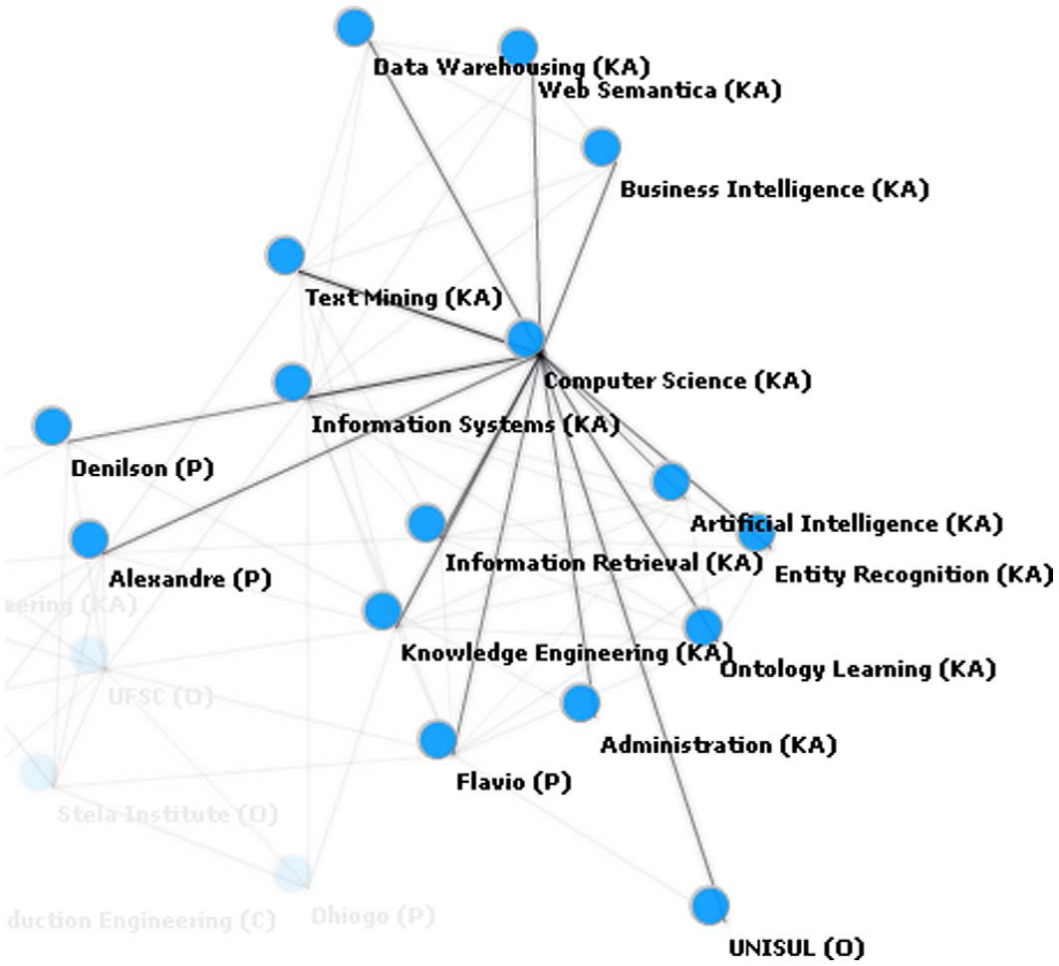
Specific network graphic for the computer science instance.

The network graphic can be zoomed in to focus on a single instance and its relations (Figure 3). In this example the instances “flavio”, “denilson,” and “alexandre” have relationships with “computer science,” which could be interpreted as these people having an undergraduate or graduate degree in the field. The instance “computer science” is also related to “information systems,” “artificial intelligence,” “information retrieval,” “text mining,” and “semantic web,” possibly indicating a similarity relationship among these areas.

**Figure 4:**
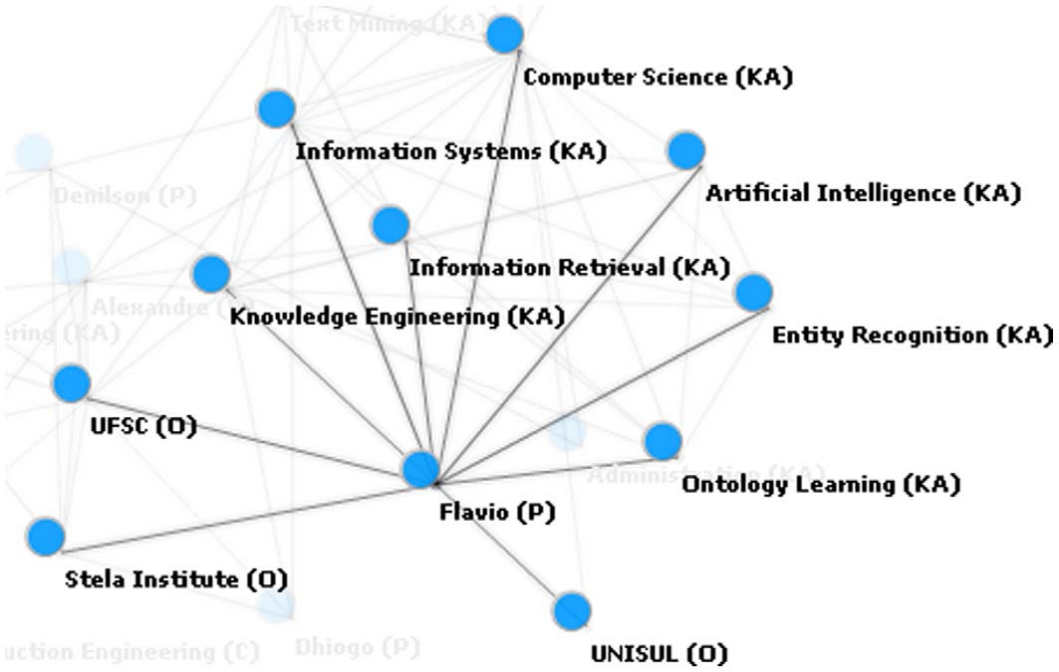
Relations among “flavio” (class person) and other instances.

Figure 4 demonstrates the relations with the instance “flavio,” identified as a person class. Relations include institutions which the knowledge engineer could manually classify as professional or academic relations. Other relations include knowledge areas such as “information retrieval,” and “entity recognition.” Additional relations with entities such as “ontology population” were not associated with a specific class, and therefore could either be manually classified by the knowledge engineer or be discarded if considered irrelevant.

**Figure 5:**
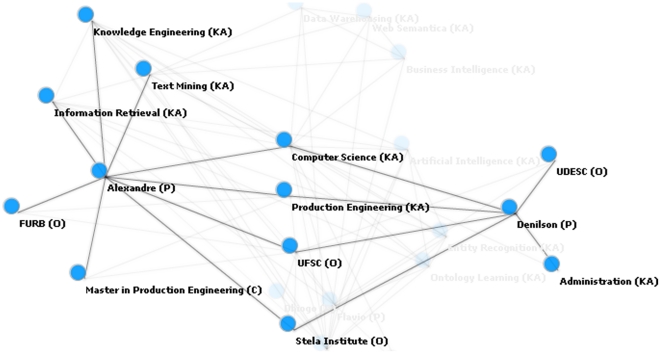
Relations between instances “denilson” and “alexandre”.

Figure 5 represents the relation between the instances of two classes of person, “denilson” and “alexandre.” Of relevance, in Table S2 “denilson” and “alexandre” do not co-occur anywhere in the text and, yet, these instances are indirectly related through other instances such as knowledge areas including “computer science” and “knowledge engineering” as well as similar institutions such as “stella institute.” A knowledge engineer could therefore infer that these instances work in the same institution and share common areas of knowledge, perhaps creating an index to demonstrate that they could also be part of the same collaborative group.

### Experimental section

In order to evaluate the scalability of this model, the study case was expanded for a larger number of CVs (curriculum vitae). The following sections describe the steps implemented to create the corpus. First, we used a group of CVs from the Lattes Platform [17]. Specifically, we included the first 1,000 CVs having the largest proportion of terms “biotechnology” in the fields related to science and technology productivity, professional activities, and projects. Retrieved CVs were stored in a relational database. This corpus was then used to extract a set of entities (class and respective description). For each individual CV, we then generated a list of classes along with their respective positioning within the text. The process to generate correlation indices involves the analysis of the co-occurrences of entities for each vector within the corpus to establish a set of relations. The resulting entity-entity correlation matrix was then generated, with correlation values determined by the degree of relationship between them. From the correlation matrix, a network can be drawn based on the choice of a specific entity. From the correlation, we then projected a network by choosing a specific entity. From this entity, we then plotted the network by choosing a factor such as ‘maximum number of connections a given node can have’ as well as ‘the specific number of levels the network should depict’. Level indicates the network depth.

**Figure 6:**
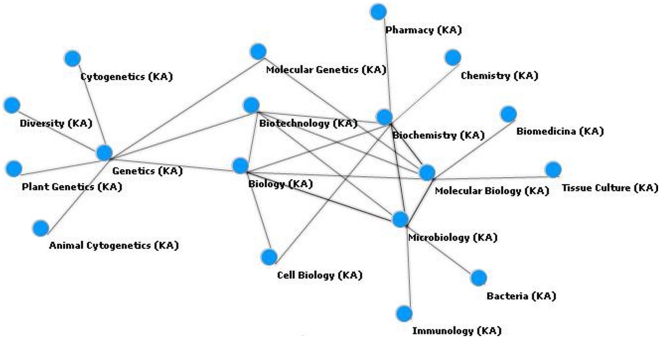
Specific network graphic for the biotechnology instance.

Figure 6 represents the main relations obtained from the concept “biotechnology.” Among these concepts are “Biology,” “Molecular Biology,” “Microbiology,” “Biochemistry,” and “Genetics.” To simplify visualization, this specific example was created with a maximum of five distinct relationships in each level of the network. Increasing the number of nodes per level, other relations are now displayed including “Engineering,” “Medicine,” “Chemistry,” “Cellular Biology,” and “Nutrition Sciences.” These concepts are, therefore, instances in the ontology related to the concept of “Biotechnology.” Each concept represents an instance and is related to other concepts. This projection allows for dense graphs with multiple connections. Our visualization approach minimizes this effect so that concepts that are less connected at a given level might be more connected at a different level. This can be verified for the concept “Chemistry,” which although associated with “Biotechnology” has a greater degree of connectivity with the second level of this network.

**Figure 7:**
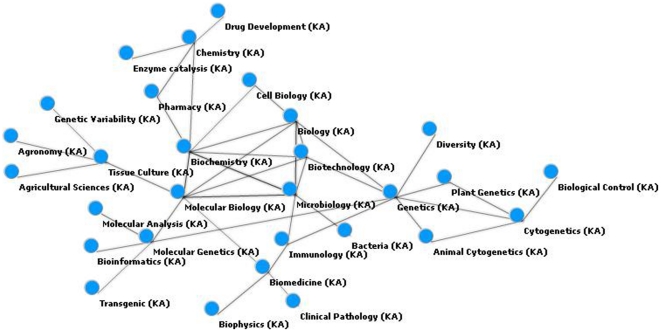
Expanded network graphic for the biotechnology instance.

The following network (Figure 7) augments the previous view focused on the “Biotechnology” concept, allow for the display of relevant connections displayed at the same level in conjunction with existing connections with the main node at a given level. This can be verified through the the connection between “Biochemistry” and “Microbiology.”

**Figure 8:**
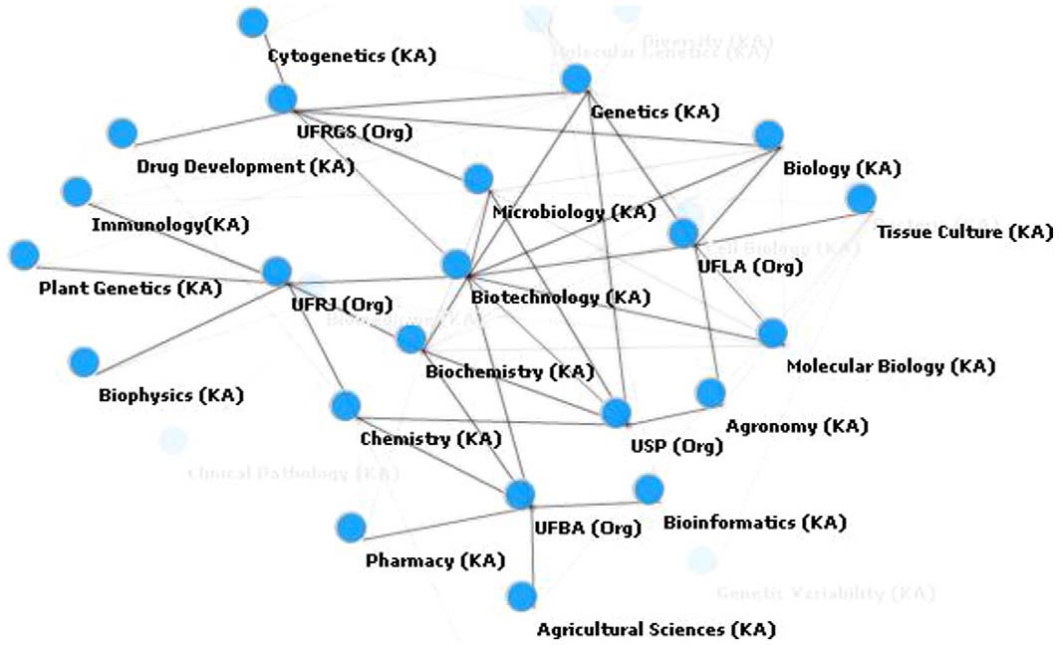
Network graphic based on knowledge area (KA) and organization (Org) entities for the biotechnology instance.

Figure 8 represents a network that expands the “Biotechnology” projection, adding entities of the type organization. Among the main extracted organizations that relate to the central concept are the acronyms for Brazilian universities, namely USP (University of Sao Paulo), UFRGS (Federal University of Rio Grande do Sul), UFLA (Federal University of Lavras), UFBA (Federal University of Bahia), and UFRJ (Federal University of Rio de Janeiro). To facilitate visualization, we only display nodes directly connected to “Biotechnology” (5 areas and organizations) and nodes connected to the first level with these organizations. Taking as an example the USP institution, the relation among Biotechnology, Genetics, Microbiology, Biochemistry, Agronomy, and Chemistry. When focusing on UFRGS, the focus is now placed on Cytogenetics, Genetics, Microbiology, Biology, and Drug Development. Another possibility of analysis is from the perspective of areas that connect two or more organizations. This is the case of Genetics, which allow the indirect connection among the institutions USP, UFRGS and UFLA.

## Discussion

To our knowledge, our study is the first to describe a novel combination of gazetteers for named-entity recognition, the LINGO algorithm for labeling cluster instances, and social network data sets for semi-automated discovery of classes and relations in a scientific domain. We have presented the utilization of a system that can identify entities to assist in the process of knowledge extraction and representation that will support ontology maintenance. Our study case and experimental study demonstrated how this technique can extract relationships among classes and their instances. In table 2, we compare our method to previous work having as its objective the automated and semi-automated maintenance and instantiation of computational ontologies.

**Table 2:** Comparison of our method with previous works.

Frameworks	Main characteristics	Main differences in relation to our project
A Flexible Framework to Experiment with Ontology Learning Techniques [18]	Semi-automated method using NLP	Requires an annotated corpus for entity recognition
A Hybrid Approach for Taxonomy Learning from Text [19]	Linguistic patterns associated with statistical reasoning	Based on statistical reasoning
Advancing Topic Ontology Learning through Term Extraction [20]	Semi-automated based on node extraction	Does not make use of collaborative databases for discovery, validation and classification of entities
Automated Ontology Learning and Validation Using Hypothesis Testing [21]	Hypothesis-driven	Hypotheses are compared against indicators retrieved from the Web
OntoLearn, a methodology for automatic learning of domain ontologies [22]	Automated extraction	Error rates related to the database, language dependent
Text2Onto - A Framework for Ontology Learning and Data-Driven Change Discovery [23]	Probabilistic Ontology Models and identification of change in data patterns	Does not require a pre-built ontology

Future work should focus on three main points. First, we will improve upon the model to identify the identification of factual relations, using resources beyond the co-occurrence model by using a semantic analysis to assist in the relation identification. Second, we will improve upon the connection with collaborative databases used for validation, specifically implementing methods that might allow us to measure its precision and ease of use. Third, we will focus on the practical use of this technology in applications that include the location of specialists, identification of skill gaps that might be important for strategic planning.

## Supporting Information

Appendix S1 **Details about BALIE extension and gazetter creation.**
(DOC)

Table S1 **Steps involved in the co-occurrence method.**
(DOCX)

Table S2 **Text example.**
(DOCX)
