# Supplementary material for: Turning Text into Research Networks: Information Retrieval and Computational Ontologies in the Creation of Scientific Databases
Source: PLoS One. 2012 Jan 3;7(1):e27499. doi: 10.1371/journal.pone.0027499 (PMC3250392; doi:10.1371/journal.pone.0027499)
Supplement: Appendix S1 — Details about BALIE extension and gazetter creation. (DOC) [file pone.0027499.s001.doc]

**Appendix S1**

**Details about BALIE extension and gazetter creation**

Para atender ao requisito de identificação do idioma português do Brasil, BALIE provê classes Java exclusivas para serem estendidas. Basicamente a classe ca.uottawa.balie.LanguageSpecific deve ser herdada, e consequentemente dois métodos devem ser implementados: o método GetAbbreviations, usado para retornar um conjunto de abreviações empregadas nas heurísticas; e, o método Decompound, empregado para quebrar o documento em tokens ou palavras. A decomposição do documento em tokens, optou-se pelo caractere de espaço em branco como único separador. A classe LanguageSpecificPortuguese foi criada para cumprir esse requisito.  Tecnicamente, BALIE possui em seu classpath uma pasta denominada lexicon com os arquivos no formato de texto (extensão txt), onde estão localizados os termos do dicionário. Para funcionar corretamente, todos os termos devem estar em minúsculo e sem quaisquer caracteres especiais, tais quais acentos e cedilha. BALIE já possui uma lista de termos classificados, como cidades, tempo (meses, feriados, dias de semana, etc.), pessoas e organizações, às quais se adicionaram os termos da Plataforma Lattes. Apenas um novo arquivo-texto contendo as áreas de conhecimento foi adicionado ao BALIE. Para conduzir a leitura do dicionário, é necessário modificar as seguintes classes Java: LexiconOnDisk, adicionando à nova lista de termos, no caso a lista de áreas de conhecimento; e NamedEntityTypeEnum, enumerando o novo tipo criado.  Após efetuar essas alterações no código-fonte, a ferramenta já se encontra preparada para extrair as entidades a partir do texto.
