# Supplementary material for: Turning Text into Research Networks: Information Retrieval and Computational Ontologies in the Creation of Scientific Databases
Source: PLoS One. 2012 Jan 3;7(1):e27499. doi: 10.1371/journal.pone.0027499 (PMC3250392; doi:10.1371/journal.pone.0027499)
Supplement: Table S1 — Steps involved in the co-occurrence method. (DOCX) [file pone.0027499.s002.docx]

**Table S1-** Steps involved in the co-occurrence method

| 1. Named-entity recognition and clustering using a combination of gazetteers and the LINGO algorithm  2. Entity frequency counts  3. Entity validation making use of social network data sets  4. Determination of entity relations through co-occurrence, defined as the number of windows (words) between any two entities along with their frequency  5. Results presentation through either instantiation of an ontology or creation of a graph network |
| --- |
