# Supplementary material for: Turning Text into Research Networks: Information Retrieval and Computational Ontologies in the Creation of Scientific Databases
Source: PLoS One. 2012 Jan 3;7(1):e27499. doi: 10.1371/journal.pone.0027499 (PMC3250392; doi:10.1371/journal.pone.0027499)
Supplement: Table S2 — Text example. (DOCX) [file pone.0027499.s003.docx]

**Table S2 - Text example**

| Flávio Ceci completed his undergraduate degree in Computer Sciences at the Federal University of Santa Catarina in 2007.  Flávio is a MSc student with a focus on Knowledge Management and Engineering at the Federal University of Santa Catarina.  He is currently a software engineer at the Stela Institute, having completed six software packages and one technical item.  Between 2004 and 2007, he participated in four research projects.  He currently participates in three research projects.  Flávio works in the Computer Sciences field, with a concentration on Information Systems, focusing primarily on entity recognition, artificial intelligence methods applied to knowledge engineering, ontology instantiation, and knowledge discovery in free text and information retrieval.  During these activities he established collaborations with 13 individuals in research manuscripts. Alexandre Leopoldo Gonçalves has an undergraduate degree in Computer Sciences by the Regional University Foundation of Blumenau (1997), Masters of Science in Production Engineering by the Federal University of Santa Catarina (2000) and a PhD in Production Engineering by the Federal Univerisity of Santa Catarina (2006).  Currently, Alexandre is a collaborator and director of the Product Unity in the Stela Institute.  Alexandre has working experience in the field of Computer Science, with a concetration on Knowledge Engineering, focusing primarily on information retrieval and extraction, text mining, and knowledge engineering.  He also has articles published in international journals, presentations in national and international conferences, and has developed software packages. Since 2001 he participates in the execution and coordination of research projects in Brazil and internationally.  Denilson Sell obtained a PhD degree in Knowledge Engineering by the Federal University of Santa Catarina in 2007.  Currently, Denilson is a faculty member at the Federal University of Santa Catarina, is a systems analyst for the Stela Institute and faculty member at the State University of Santa Catarina.  He has published one peer-reviewed article and 16 conference abstracts. He has created 16 software packages, and 11 technical reports. He participated in 3 international conferences and 6 conferences in Brazil.  Denilson co-mentored 5 Master theses and 2 undergraduate theses in Computer Sciences and Business Administration. He received two awards. Between 1997 and 2005 he participated in 11 research projects. He currently participates in 5 research projects, coordinating 2 of these. He works in the field of Computer Sciences, with an emphasis in Information Systems. In his professional activities he interacted with 55 collaborators in co-authorships of scientific projects. Dhiogo Cardoso da Silva has an undergraduate degree in Information Systems by the Federal University of Santa Catarina (2007) and is currently a Masters student in Knowledge Engineering by the Federal University of Santa Catarina. Currently, Dhiogo is a collaborator at the Stela Institute. Dhiogo has experience in the field of Computer Science, with an emphasis in Information Systems,  focusing on Business Intelligence, Semantic Web, Data Warehousing, and Text Mining. |
| --- |
